# 2-(2-Chloro­phen­oxy)benzoic acid

**DOI:** 10.1107/S1600536811006301

**Published:** 2011-03-02

**Authors:** Lu Shi, Qin Zhang, Qi Xiao, Tao Wu, Hong-Jun Zhu

**Affiliations:** a Department of Applied Chemistry, College of Science, Nanjing University of Technology, Nanjing 210009, People’s Republic of China

## Abstract

In the crystal structure of the title compound, C_13_H_9_ClO_3_, the mol­ecules form classical O—H⋯O hydrogen-bonded carb­oxy­lic acid dimers. These dimers are linked by C—H⋯π inter­actions into a three-dimensional network. The benzene rings are oriented at a dihedral angle of 77.8 (1)°.

## Related literature

For applications of the title compound, see: Yang *et al.* (1972 ▶). For a related structure, see: Parkin *et al.* (2005 ▶). For the synthesis of the title compound, see: Rolando *et al.* (1995 ▶). For bond-length data, see: Allen *et al.* (1987 ▶).
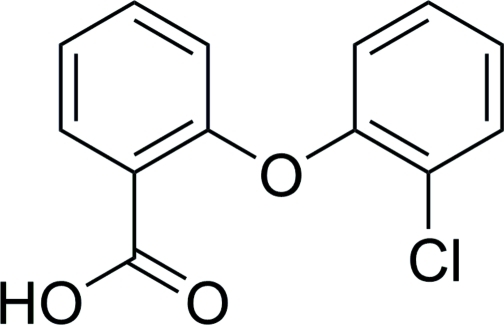


## Experimental

### 

#### Crystal data

C_13_H_9_ClO_3_
*M* *_r_* = 248.65
Monoclinic, 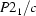

*a* = 6.9930 (14) Å
*b* = 24.986 (5) Å
*c* = 7.5140 (15) Å
β = 115.79 (3)°
*V* = 1182.1 (4) Å^3^
*Z* = 4
Mo *K*α radiation
μ = 0.32 mm^−1^
*T* = 293 K
0.30 × 0.20 × 0.10 mm

#### Data collection

Enraf–Nonius CAD-4 diffractometer
Absorption correction: ψ scan (North *et al.*, 1968 ▶) *T*_min_ = 0.912, *T*_max_ = 0.969
4651 measured reflections
2177 independent reflections
1061 reflections with *I* > 2σ(*I*)
*R*_int_ = 0.088
3 standard reflections every 200 reflections  intensity decay: 1%

#### Refinement

*R*[*F* ^2^ > 2σ(*F* ^2^)] = 0.053
*wR*(*F* ^2^) = 0.110
*S* = 1.01
2177 reflections
154 parameters
H-atom parameters constrained
Δρ_max_ = 0.20 e Å^−3^
Δρ_min_ = −0.31 e Å^−3^

### 

Data collection: *CAD-4 Software* (Enraf–Nonius, 1985 ▶); cell refinement: *CAD-4 Software*; data reduction: *XCAD4* (Harms & Wocadlo, 1995 ▶); program(s) used to solve structure: *SHELXS97* (Sheldrick, 2008 ▶); program(s) used to refine structure: *SHELXL97* (Sheldrick, 2008 ▶); molecular graphics: *SHELXTL* (Sheldrick, 2008 ▶); software used to prepare material for publication: *SHELXTL*.

## Supplementary Material

Crystal structure: contains datablocks I, global. DOI: 10.1107/S1600536811006301/bq2280sup1.cif

Structure factors: contains datablocks I. DOI: 10.1107/S1600536811006301/bq2280Isup2.hkl

Additional supplementary materials:  crystallographic information; 3D view; checkCIF report

## Figures and Tables

**Table 1:** Hydrogen-bond geometry (Å, °) *Cg*1 is the centroid of C1–C6 ring.

*D*—H⋯*A*	*D*—H	H⋯*A*	*D*⋯*A*	*D*—H⋯*A*
O2—H2*B*⋯O3^i^	0.82	1.81	2.621 (3)	172
C11—H11*A*⋯*Cg*1^ii^	0.93	2.74	3.582 (4)	151

Symmetry codes: (i) 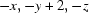; (ii) 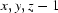.

**Fig. 1.**
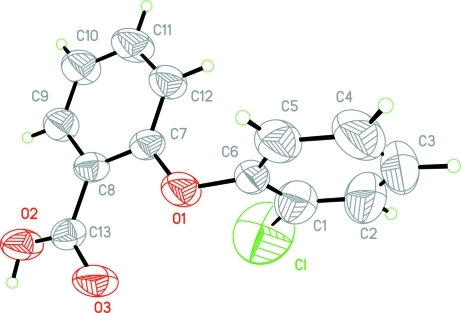
The molecular structure of (I), with the atom-numbering scheme. Displacement ellipsoids are drawn at the 50% probability level.

**Fig. 2.**
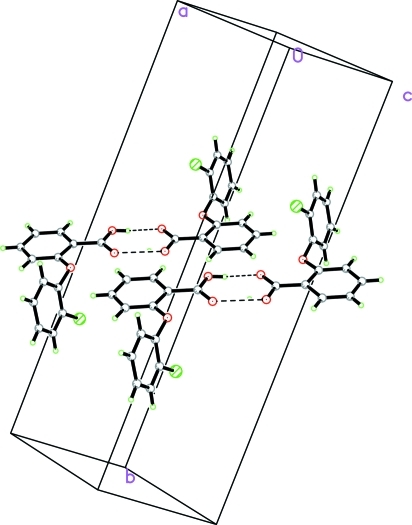
A packing diagram of (I). Hydrogen bonds are shown as dashed lines.

## supplementary crystallographic information

### Comment

The tittle compound, 2-(2-chlorophenoxy)benzoic acid is an important intermediate (Yang *et al.*, 1972). And we report here the crystal structure of the title compound, (I).

The molecular structure of (I) is shown in Fig. 1, and the intermolecular O—H···O hydrogen bond (Table 1) results in the formation of carboxylic acid dimers (Fig. 2.). The bond lengths and angles are within normal ranges (Allen *et al.*, 1987).

In the molecule of (I), the dihedral angle of the rings (C1—C6) and (C7—C12) is 77.8 (1)°. The O atom lies in the bonded benzene ring planes, and Cl atom is connected with the phenyl ring (C1—C6).

In the crystal of (I), the molecules were connected together *via* O—H···O intermolecular hydrogen bonds to form dimers. These dimers are linked by C—H···π interactions to give a three-dimensional network, which seems to be very effective in the stabilization of the crystal structure.

### Experimental

The title compound, (I) was prepared by the method of Ullmann condensation reaction reported in literature (Rolando *et al.*, 1995). The crystals were obtained by dissolving (I) (0.2 g, 0.8 mmol) in ethanol (25 ml) and evaporating the solvent slowly at room temperature for about 5 d.

### Refinement

H atoms were positioned geometrically and refined as riding groups, with O—H = 0.82 and C—H = 0.93 Å for aromatic H, and constrained to ride on their parent atoms, with *U*_iso_(H) = *xU*_eq_(C), where *x* = 1.2 for aromatic H, and *x* = 1.5 for other H.

### Crystal data

**Table:**

C_13_H_9_ClO_3_	*F*(000) = 512
*M**_r_* = 248.65	*D*_x_ = 1.397 Mg m^−^^3^
Monoclinic, *P*2_1_/*c*	Mo *K*α radiation, λ = 0.71073 Å
Hall symbol: -P 2ybc	Cell parameters from 25 reflections
*a* = 6.9930 (14) Å	θ = 9–14°
*b* = 24.986 (5) Å	µ = 0.32 mm^−^^1^
*c* = 7.5140 (15) Å	*T* = 293 K
β = 115.79 (3)°	Block, colourless
*V* = 1182.1 (4) Å^3^	0.30 × 0.20 × 0.10 mm
*Z* = 4	

### Data collection

**Table:**

Enraf–Nonius CAD-4 diffractometer	1061 reflections with *I* > 2σ(*I*)
Radiation source: fine-focus sealed tube	*R*_int_ = 0.088
graphite	θ_max_ = 25.4°, θ_min_ = 1.6°
ω/2θ scans	*h* = 0→8
Absorption correction: ψ scan (North *et al.*, 1968)	*k* = −30→30
*T*_min_ = 0.912, *T*_max_ = 0.969	*l* = −9→8
4651 measured reflections	3 standard reflections every 200 reflections
2177 independent reflections	intensity decay: 1%

### Refinement

**Table:**

Refinement on *F*^2^	Primary atom site location: structure-invariant direct methods
Least-squares matrix: full	Secondary atom site location: difference Fourier map
*R*[*F*^2^ > 2σ(*F*^2^)] = 0.053	Hydrogen site location: inferred from neighbouring sites
*wR*(*F*^2^) = 0.110	H-atom parameters constrained
*S* = 1.01	*w* = 1/[σ^2^(*F*_o_^2^) + (0.035*P*)^2^] where *P* = (*F*_o_^2^ + 2*F*_c_^2^)/3
2177 reflections	(Δ/σ)_max_ < 0.001
154 parameters	Δρ_max_ = 0.20 e Å^−^^3^
0 restraints	Δρ_min_ = −0.31 e Å^−^^3^

### Special details

**Table:**

Geometry. All e.s.d.'s (except the e.s.d. in the dihedral angle between two l.s. planes) are estimated using the full covariance matrix. The cell e.s.d.'s are taken into account individually in the estimation of e.s.d.'s in distances, angles and torsion angles; correlations between e.s.d.'s in cell parameters are only used when they are defined by crystal symmetry. An approximate (isotropic) treatment of cell e.s.d.'s is used for estimating e.s.d.'s involving l.s. planes.
Refinement. Refinement of *F*^2^ against ALL reflections. The weighted *R*-factor *wR* and goodness of fit *S* are based on *F*^2^, conventional *R*-factors *R* are based on *F*, with *F* set to zero for negative *F*^2^. The threshold expression of *F*^2^ > σ(*F*^2^) is used only for calculating *R*-factors(gt) *etc*. and is not relevant to the choice of reflections for refinement. *R*-factors based on *F*^2^ are statistically about twice as large as those based on *F*, and *R*- factors based on ALL data will be even larger.

### Fractional atomic coordinates and isotropic or equivalent isotropic displacement parameters (Å^2^)

**Table:**

	*x*	*y*	*z*	*U*_iso_*/*U*_eq_	
Cl	0.30495 (19)	0.80926 (6)	0.11968 (19)	0.1627 (6)	
O1	0.5536 (3)	0.90532 (7)	0.1714 (2)	0.0654 (6)	
C1	0.5689 (5)	0.81539 (15)	0.2793 (5)	0.0820 (9)	
O2	0.0554 (3)	0.99730 (8)	−0.2082 (2)	0.0756 (6)	
H2B	−0.0220	1.0088	−0.1606	0.113*	
C2	0.6811 (8)	0.77525 (16)	0.4047 (6)	0.1038 (13)	
H2A	0.6136	0.7432	0.4050	0.125*	
O3	0.2120 (3)	0.96137 (9)	0.0872 (2)	0.0780 (7)	
C3	0.8874 (8)	0.78149 (18)	0.5273 (6)	0.1116 (16)	
H3A	0.9597	0.7542	0.6150	0.134*	
C4	0.9925 (6)	0.82717 (19)	0.5254 (5)	0.0998 (13)	
H4A	1.1367	0.8308	0.6076	0.120*	
C5	0.8814 (5)	0.86787 (13)	0.3997 (4)	0.0701 (9)	
H5A	0.9506	0.8994	0.3967	0.084*	
C6	0.6706 (5)	0.86214 (12)	0.2799 (3)	0.0533 (7)	
C7	0.5230 (4)	0.91164 (10)	−0.0195 (3)	0.0499 (7)	
C8	0.3564 (4)	0.94517 (10)	−0.1407 (3)	0.0492 (7)	
C9	0.3345 (4)	0.95491 (11)	−0.3322 (3)	0.0608 (8)	
H9A	0.2275	0.9778	−0.4143	0.073*	
C10	0.4647 (5)	0.93199 (13)	−0.4018 (4)	0.0755 (9)	
H10A	0.4463	0.9388	−0.5299	0.091*	
C11	0.6243 (5)	0.89853 (13)	−0.2802 (4)	0.0760 (10)	
H11A	0.7140	0.8826	−0.3268	0.091*	
C12	0.6529 (4)	0.88827 (11)	−0.0903 (3)	0.0619 (8)	
H12A	0.7608	0.8654	−0.0099	0.074*	
C13	0.2020 (4)	0.96877 (11)	−0.0801 (3)	0.0492 (6)	

### Atomic displacement parameters (Å^2^)

**Table:**

	*U*^11^	*U*^22^	*U*^33^	*U*^12^	*U*^13^	*U*^23^
Cl	0.1046 (9)	0.1774 (14)	0.1807 (12)	−0.0608 (9)	0.0384 (8)	−0.0066 (9)
O1	0.0724 (13)	0.0844 (15)	0.0475 (10)	0.0307 (11)	0.0338 (10)	0.0141 (9)
C1	0.089 (3)	0.074 (2)	0.091 (2)	−0.002 (2)	0.046 (2)	0.0038 (19)
O2	0.0668 (14)	0.1086 (17)	0.0618 (11)	0.0366 (12)	0.0375 (11)	0.0274 (11)
C2	0.133 (4)	0.078 (3)	0.114 (3)	0.014 (3)	0.066 (3)	0.027 (2)
O3	0.0779 (14)	0.1155 (18)	0.0523 (11)	0.0450 (13)	0.0392 (10)	0.0240 (10)
C3	0.139 (4)	0.113 (4)	0.095 (3)	0.071 (3)	0.062 (3)	0.047 (3)
C4	0.073 (3)	0.146 (4)	0.076 (2)	0.045 (3)	0.028 (2)	0.018 (3)
C5	0.055 (2)	0.088 (3)	0.0663 (18)	0.0113 (18)	0.0260 (16)	0.0073 (17)
C6	0.0579 (18)	0.063 (2)	0.0439 (14)	0.0175 (17)	0.0267 (14)	0.0095 (13)
C7	0.0558 (17)	0.0591 (18)	0.0421 (14)	0.0042 (14)	0.0283 (13)	0.0022 (12)
C8	0.0453 (15)	0.0626 (19)	0.0420 (14)	0.0029 (14)	0.0210 (13)	0.0000 (12)
C9	0.0648 (19)	0.072 (2)	0.0479 (15)	0.0127 (16)	0.0267 (15)	0.0067 (13)
C10	0.091 (2)	0.096 (2)	0.0515 (17)	0.028 (2)	0.0423 (18)	0.0130 (16)
C11	0.088 (2)	0.100 (3)	0.0565 (17)	0.026 (2)	0.0459 (17)	−0.0012 (17)
C12	0.066 (2)	0.074 (2)	0.0525 (16)	0.0186 (16)	0.0320 (15)	0.0053 (14)
C13	0.0425 (15)	0.0625 (18)	0.0423 (14)	0.0026 (14)	0.0181 (12)	0.0064 (13)

### Geometric parameters (Å, °)

**Table:**

Cl—C1	1.716 (3)	C5—C6	1.360 (3)
O1—C7	1.364 (3)	C5—H5A	0.9300
O1—C6	1.385 (3)	C7—C12	1.367 (3)
C1—C2	1.366 (4)	C7—C8	1.402 (3)
C1—C6	1.367 (4)	C8—C9	1.401 (3)
O2—C13	1.276 (3)	C8—C13	1.466 (3)
O2—H2B	0.8200	C9—C10	1.358 (4)
C2—C3	1.341 (5)	C9—H9A	0.9300
C2—H2A	0.9300	C10—C11	1.376 (4)
O3—C13	1.242 (2)	C10—H10A	0.9300
C3—C4	1.361 (5)	C11—C12	1.376 (3)
C3—H3A	0.9300	C11—H11A	0.9300
C4—C5	1.375 (4)	C12—H12A	0.9300
C4—H4A	0.9300		
			
C7—O1—C6	119.41 (19)	O1—C7—C8	117.3 (2)
C2—C1—C6	118.9 (3)	C12—C7—C8	120.5 (2)
C2—C1—Cl	122.5 (3)	C9—C8—C7	117.4 (2)
C6—C1—Cl	118.6 (3)	C9—C8—C13	118.9 (2)
C13—O2—H2B	109.5	C7—C8—C13	123.7 (2)
C3—C2—C1	120.8 (4)	C10—C9—C8	122.0 (3)
C3—C2—H2A	119.6	C10—C9—H9A	119.0
C1—C2—H2A	119.6	C8—C9—H9A	119.0
C2—C3—C4	121.0 (4)	C9—C10—C11	119.1 (2)
C2—C3—H3A	119.5	C9—C10—H10A	120.4
C4—C3—H3A	119.5	C11—C10—H10A	120.4
C3—C4—C5	118.8 (4)	C10—C11—C12	120.8 (3)
C3—C4—H4A	120.6	C10—C11—H11A	119.6
C5—C4—H4A	120.6	C12—C11—H11A	119.6
C6—C5—C4	120.2 (3)	C7—C12—C11	120.2 (2)
C6—C5—H5A	119.9	C7—C12—H12A	119.9
C4—C5—H5A	119.9	C11—C12—H12A	119.9
C5—C6—C1	120.3 (3)	O3—C13—O2	121.3 (2)
C5—C6—O1	120.0 (3)	O3—C13—C8	122.0 (2)
C1—C6—O1	119.4 (3)	O2—C13—C8	116.7 (2)
O1—C7—C12	122.2 (2)		
			
C6—C1—C2—C3	0.3 (5)	O1—C7—C8—C9	175.4 (2)
Cl—C1—C2—C3	179.9 (3)	C12—C7—C8—C9	−2.2 (4)
C1—C2—C3—C4	−2.4 (6)	O1—C7—C8—C13	−6.7 (4)
C2—C3—C4—C5	2.2 (6)	C12—C7—C8—C13	175.8 (2)
C3—C4—C5—C6	−0.1 (5)	C7—C8—C9—C10	1.7 (4)
C4—C5—C6—C1	−1.8 (4)	C13—C8—C9—C10	−176.4 (3)
C4—C5—C6—O1	172.1 (2)	C8—C9—C10—C11	−0.5 (5)
C2—C1—C6—C5	1.7 (4)	C9—C10—C11—C12	−0.1 (5)
Cl—C1—C6—C5	−177.9 (2)	O1—C7—C12—C11	−175.8 (3)
C2—C1—C6—O1	−172.3 (3)	C8—C7—C12—C11	1.6 (4)
Cl—C1—C6—O1	8.1 (4)	C10—C11—C12—C7	−0.4 (5)
C7—O1—C6—C5	95.1 (3)	C9—C8—C13—O3	179.5 (3)
C7—O1—C6—C1	−90.9 (3)	C7—C8—C13—O3	1.7 (4)
C6—O1—C7—C12	−21.0 (4)	C9—C8—C13—O2	−0.2 (4)
C6—O1—C7—C8	161.5 (2)	C7—C8—C13—O2	−178.1 (2)

### Hydrogen-bond geometry (Å, °)

**Table:**

Cg1 is the centroid of C1–C6 ring.

**Table:**

*D*—H···*A*	*D*—H	H···*A*	*D*···*A*	*D*—H···*A*
O2—H2B···O3^i^	0.82	1.81	2.621 (3)	172
C11—H11A···Cg1^ii^	0.93	2.74	3.582 (4)	151

Symmetry codes: (i) −*x*, −*y*+2, −*z*; (ii) *x*, *y*, *z*−1.
